# Bibliometric and visual analysis of chemotherapy-induced nausea and vomiting (2004-2023)

**DOI:** 10.3389/fonc.2024.1377486

**Published:** 2024-04-24

**Authors:** Shao-Chuang Tian, Jing Yang, Xin Li, Rong-Xia Huang, Jian Chen

**Affiliations:** ^1^ Department of Oncology, The First People’s Hospital of Kunming, Kunming, China; ^2^ Department of Gynecology, Kunming Maternal and Child Health Hospital, Kunming, China

**Keywords:** bibliometrics, chemotherapy-induced nausea and vomiting, CiteSpace, VOSviewers, CINV

## Abstract

**Background:**

Patients undergoing chemotherapy often encounter troubling and common side effects, notably Chemotherapy-induced nausea and vomiting (CINV). This side effect not only impairs the patient’s quality of life but could also result in the interruption or discontinuation of the chemotherapy treatment. Consequently, research into CINV has consistently remained a focal point in the realm of clinical medicine. In this research domain, bibliometric analysis has not been conducted. The purpose of this study is to deliver a thorough summary of the knowledge framework and key areas of interest in the field of Chemotherapy-induced nausea and vomiting, using bibliometric methods. This approach aims to furnish novel concepts and pathways for investigators working in this area.

**Methods:**

Publications focusing on Chemotherapy-induced nausea and vomiting, spanning from 2004 to 2023, were identified using the Web of Science Core Collection (WoSCC) database. Tools such as VOSviewer, CiteSpace, and the R package “bibliometrix” were employed for this bibliometric analysis.

**Results:**

This research covers 734 publications from 61 countries, with the United States and China being the primary contributors. There has been a significant rise in the volume of papers published in the most recent decade compared to the one before it, spanning over the past twenty years. However, the annual publication rate in the last ten years has not shown a significant upward trend. The University of Toronto, Merck & Co., Sun Yat-sen University, and Helsinn Healthcare SA emerged as the principal research institutions in this field. Supportive Care in Cancer stands out as the most frequently published and cited journal in this domain. These works are contributed by 3,917 authors, with Rudolph M Navari, Matti Aapro, Shimokawa Mototsugu, and Lee Schwartzberg being among those who have published the most. Paul J. Hesketh is notably the most co-cited author. The primary focus of this research field lies in exploring the mechanisms of CINV and the therapeutic strategies for managing it. Key emerging research hotspots are represented by terms such as “Chemotherapy-induced nausea and vomiting,” “nausea,” “vomiting,” “chemotherapy,” and “antiemetics.”

**Conclusion:**

This represents the inaugural bibliometric study to thoroughly outline the research trends and advancements in the field of CINV. It highlights the latest research frontiers and trending directions, offering valuable insights for scholars engaged in studying CINV.

## 1 Introduction

Chemotherapy-induced nausea and vomiting (CINV) rank as some of the most distressing adverse effects experienced by patients undergoing chemotherapy (1, 2). CINV encompasses various types, such as acute, delayed, breakthrough, refractory, and anticipatory nausea and vomiting (3, 4). The underlying pathophysiology of CINV remains incompletely understood. Current understanding posits vomiting as a multi-step reflex process regulated by the vomiting center (5). The mechanisms involved primarily encompass peripheral and central pathways: (1) Peripheral pathway: Anticancer drugs trigger the release of 5-HT3 from enterochromaffin cells located in the gastrointestinal mucosa. This release leads to the activation of 5-HT3 receptors, commonly causing acute vomiting (5); (2) Central pathway: Substance P induces delayed vomiting by interacting with neurokinin 1 (NK-1) receptors found in the brain’s vomiting center. (5). Although nausea and vomiting are mechanistically interrelated, they may involve distinct neuro-transmission pathways (6), with nausea often occurring at a higher frequency than vomiting.

Despite significant advancements in the prevention of CINV, as many as 40% of cancer patients continue to suffer from symptoms like nausea and vomiting post-chemotherapy, which drastically affects their life quality (3, 7). Untimely management of CINV can reduce patient adherence to chemotherapy regimens, thereby compromising treatment efficacy (8, 9). Consequently, identifying more effective strategies for managing CINV is crucial for successful treatment outcomes.

Thankfully, as a developing field, bibliometrics enables the examination of contributions from various countries/regions, institutions, authors, and journals within a specific area. This approach yields valuable insights into the hotspots of research and emerging keywords. Moreover, bibliometrics can forecast future developmental trends in the field (10). Commonly utilized bibliometric tools such as CiteSpace (11), VOSviewer (12), and the R software package “bibliometrix” (13) are instrumental in visualizing the outcomes of literature analysis. These tools have seen extensive application in medical fields, including oncology (14) and orthopedics (15).

Research on CINV has been a focal point in clinical medicine. However, this area has not yet been subjected to bibliometric analysis. By conducting a bibliometric analysis of CINV-related literature over the past two decades (2004 to 2023), Our objective was to determine the major contributors and assess the present state of research, unearth the latest findings and hotspots within the field, and anticipate upcoming research trends and potential developments in this domain.

## 2 Methods

### 2.1 Search strategy

Relative to other pertinent databases, data sourced from the Web of Science (WoS) are deemed more representative and influential. Additionally, it has been demonstrated that bibliometric analyses utilizing WoS data can be visualized with greater effectiveness (16, 17).

**Figure 1:**
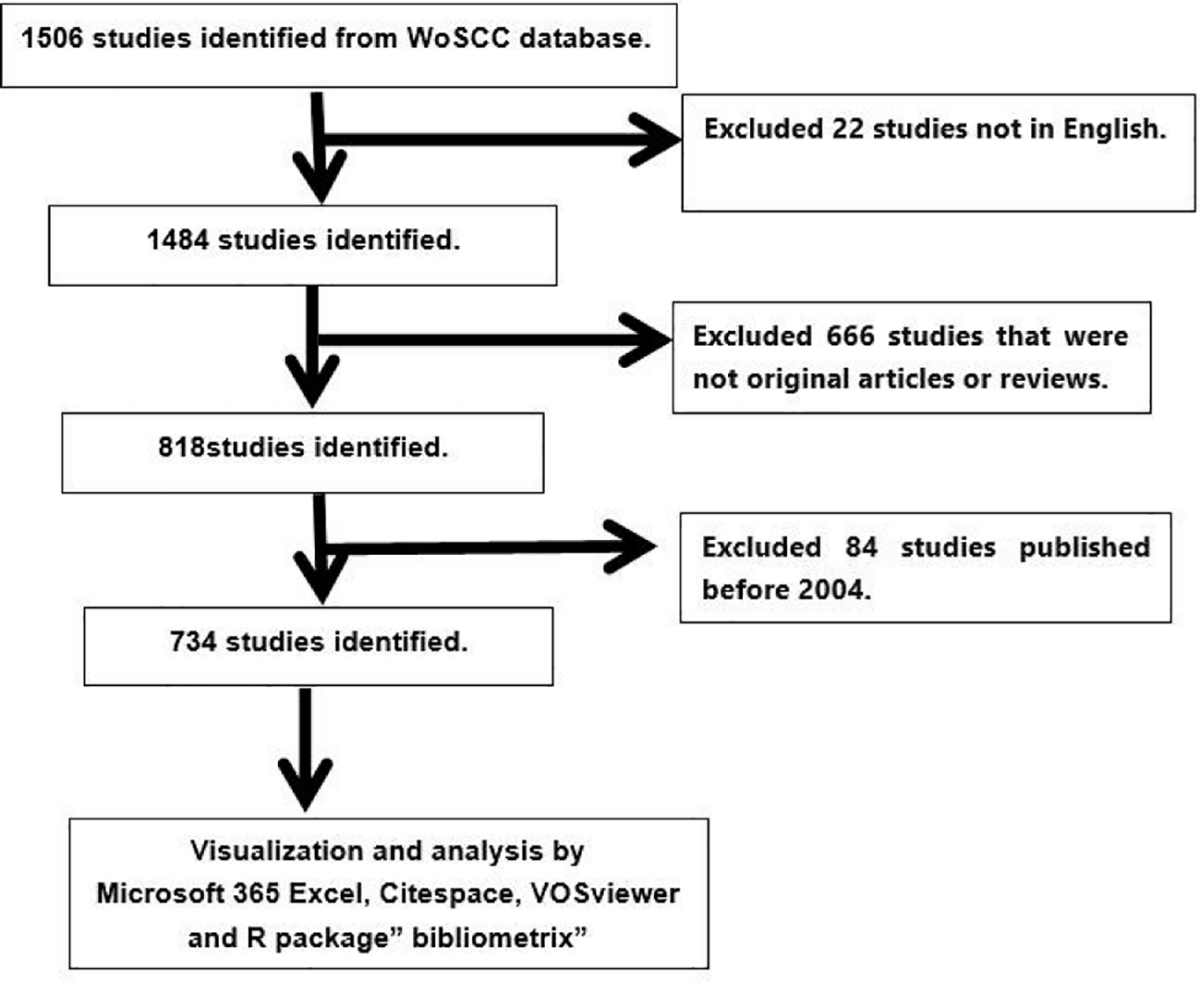
Publications screening flowchart.

On October 5, 2023, a literature search was performed in the Web of Science Core Collection (WoSCC) database, accessible at https://www.webofscience.com/wos/WoSC/basic search. The search query was “TI= (Chemotherapy-induced nausea and vomiting) OR (TI=CINV)”. To maintain the timeliness of the data, the publication date range was limited from January 1, 2004, to September 30, 2023. The search included only articles and reviews written in English. The gathered data was saved in a txt file, formatted to include the Full Record and Cited References. Adhering to these parameters resulted in a total of 734 publications, which were then used for further visualization analysis (**Figure 1**).

### 2.2 Data analysis

VOSviewer (version 1.6.19) is a software designed for bibliometric analysis, adept at extracting essential information from a large number of publications (12). It’s commonly employed for mapping collaboration, co-citation, and co-occurrence networks (18). In our study, VOSviewer was mainly used for analyzing countries and institutions, journals and co-cited journals, authors, and co-cited authors, as well as keyword co-occurrence. In the VOSviewer-generated maps, nodes symbolize entities like countries, institutions, journals, and authors, and it should be noted that we merged England, Scotland, Wales, and Northern Ireland into the United Kingdom through the document replacement operation. The size and color of these nodes denote the quantity and type of the entities, respectively. The line thickness between nodes indicates the level of collaboration or co-citation (15).

CiteSpace (version 6.2.R4) is another software tool for bibliometric analysis and visualization (11, 18). In this research, CiteSpace was used to produce journal dual-overlay maps and to perform literature analysis using Citation Bursts.

The R package “bibliometrix” (version 4.1.3) (https://www.bibliometrix.org) was utilized for thematic evolution analysis and to construct a global distribution network of CINV publications (13). The quartiles and impact factors of journals were obtained from the Journal Citation Reports 2020. Additionally, Microsoft 365 Excel was employed for the quantitative analysis of the publications.

## 3 Results

### 3.1 Annual publication growth trends

**Figure 2:**
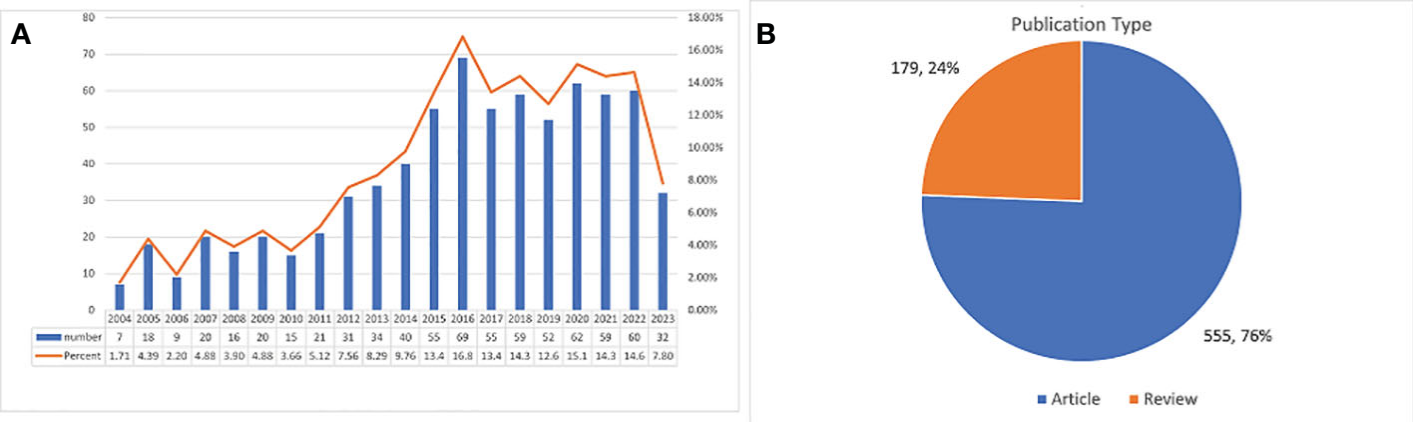
Annual growth trends of publication and the distribution of publication type.

For an in-depth analysis of the current research landscape in CINV, Microsoft 365 Excel was employed to compile and examine the annual publication count from 2004 to 2023. This data was then used to generate a trend graph. As depicted in **Figure 2A**, there is a notable uptick in the volume of papers published in the most recent decade compared to the preceding one. However, there hasn’t been a noticeable upward trend in the annual publication volume in the last ten years. This suggests that research in this field may not have received adequate attention in recent years, which could be one of the reasons for the insufficiently effective management of CINV. Therefore, CINV-related research must receive more focus and attention, highlighting the untapped potential in this area. Regarding the types of publications (**Figure 2B**), original research articles constitute a major portion (555, 75.6%), while reviews represent a smaller fraction (179, 24.4%).

### 3.2 Country and institutional analysis

Our analysis encompassed publications from 61 countries and 1,557 institutions. The top ten contributing countries span Europe, Asia, and North America, with Europe (n=4), Asia (n=3), and North America (n=2) being the primary regions (**Table 1**). The United States stands at the forefront in terms of publication volume (n=251, 34.2%), followed by China (n=125, 17.0%), Japan (n=90, 12.3%), and Italy (n=57, 7.8%). Notably, the cumulative contributions from China and the United States constitute over half of the total (51.2%).

**Table 1:** Top 10 countries and institutions on the research of CINV.

Rank	Country	Counts	Rank	Institution	Counts
1	United States	251	1	University of Toronto(Canada)	28
2	China	125	2	Merck and Co Inc (United States)	20
3	Japan	90	3	Helsinn Healthcare SA(Switzerland)	18
4	Italy	57	4	Sun Yat-Sen University (China)	18
5	Switzerland	56	5	Clinique de Genolier (Switzerland)	17
6	Canada	55	6	The Hospital for Sick Children(Canada)	16
7	Germany	45	7	West Clinic (United States)	15
8	Australia	28	8	National Cancer Center (United States)	14
9	United Kingdom	27	9	Fukuoka University (Japan)	12
10	Iran	25	9	Indiana University (United States)	12
			9	University of Vermont (United States)	12

**Figure 3:**
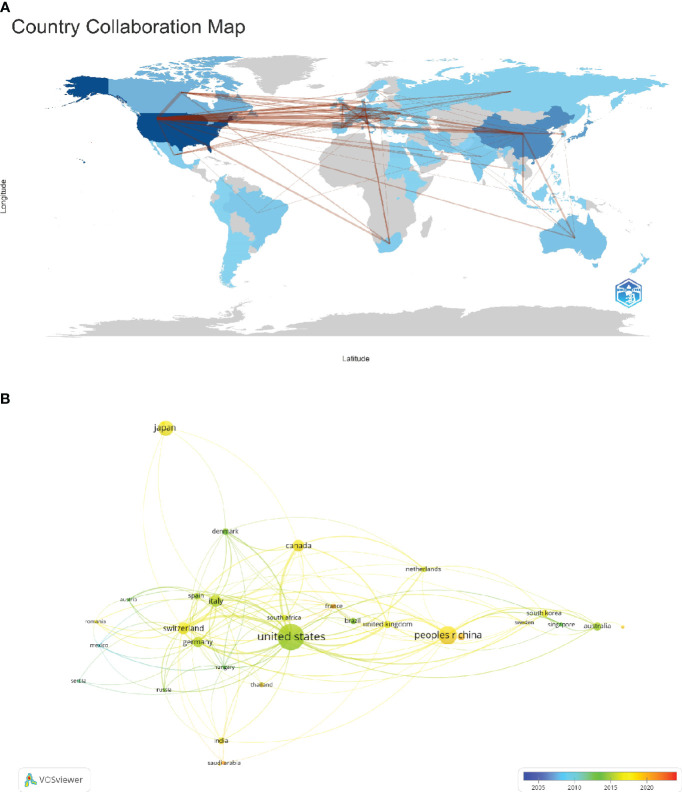
The geographical distribution and visualization of countries on the research of CINV.

Further, we focused on data from 32 countries that had published five or more papers, creating a collaborative network map based on publication counts and inter-country relationships (**Figures 3A, B**). This map highlighted the extent of international collaborations, and it should be noted that the United Kingdom in **Table 1** and **Figure 3B** is achieved by merging England, Scotland, Wales, and Northern Ireland through a document substitution operation. For example, China maintains strong collaborative ties with the United States and the United Kingdom. In a similar vein, the United States shows significant collaboration with countries like Canada, Switzerland, China, and the United Kingdom.

**Figure 4:**
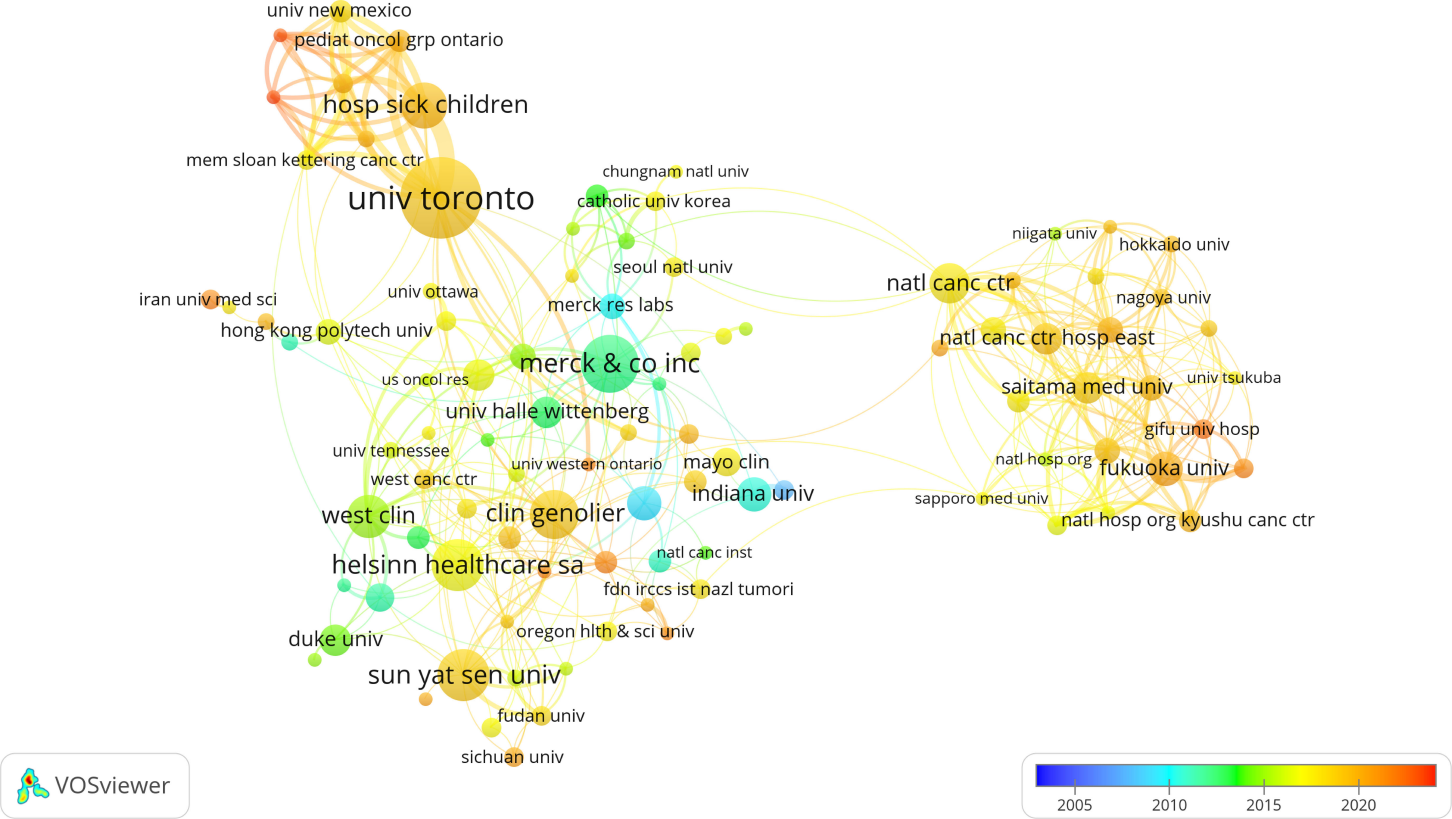
The visualization of institutions on the research of CINV.

The leading four institutions in terms of publication volume on CINV are the University of Toronto (n=28, 3.8%), Merck & Co. (n=20, 2.7%), Sun Yat-sen University (n=18, 2.5%), and Helsinn Healthcare SA (n=18, 2.5%). To further analyze institutional collaboration, we chose 100 institutions each having published a minimum of five papers. A collaborative network was then constructed, visualizing both the number of publications and the inter-institutional relationships (**Figure 4**). From **Figure 4**, it is observable that while the four institutions do not have extensive intersections and collaborations in their research, there is a considerable amount of related research surrounding each of them. Specifically, the University of Toronto has close collaborations with The Hospital for Sick Children (HSC), the Pediatric Oncology Group of Ontario (POGO), the University of New Mexico, and the Connecticut Children’s Medical Center. Merck & Co. collaborates significantly with The Medical Oncology Centre of Rosebank, Martin Luther University Halle-Wittenberg, and the University of Vermont. Sun Yat-sen University maintains tight collaborations with Fudan University, Peking University, Sichuan University, and Duke University. Additionally, Helsinn Healthcare SA has substantial collaborations with the University of California, San Francisco, and the University of Vermont.

### 3.3 Journals and co-cited journals

**Figure 5:**
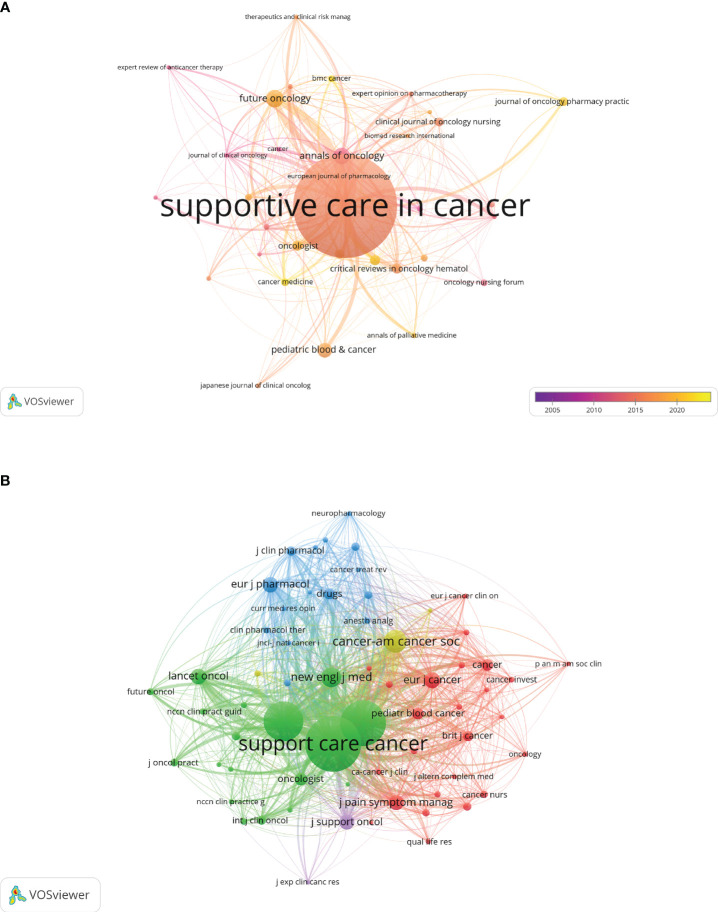
The visualization of journals and co-cited journals on the research of CINV.

CINV research has been published in 272 journals. The leading journal in terms of publication volume is ‘Supportive Care in Cancer’ (n=119, 16.2%), succeeded by ‘Future Oncology’ (n=20, 2.7%), ‘Annals of Oncology’ (n=19, 2.6%), and ‘Pediatric Blood & Cancer’ (n=6, 2.3%). Among these, ‘Annals of Oncology’ boasts the highest impact factor (IF=50.5), with ‘Critical Reviews in Oncology Hematology’ following (IF=6.2). We identified 34 journals with at least five related publications each for constructing a journal network diagram (**Figure 5A**), which reveals robust citation interactions among ‘Supportive Care in Cancer’ and others like ‘Future Oncology’, ‘Annals of Oncology’, and ‘Pediatric Blood & Cancer’.

**Table 2** highlights the top 10 co-cited journals, three of which have been cited over 1000 times. Leading in co-citations is ‘Supportive Care in Cancer’ (Co-citation=3066), with ‘Journal of Clinical Oncology’ (Co-citation=2210) and ‘Annals of Oncology’ (Co-citation=1759) following. The ‘New England Journal of Medicine’ holds the highest impact factor (IF=158.5) among these, trailed by ‘Lancet Oncology’ (IF=51.1) and ‘Annals of Oncology’ (IF=50.5). For the co-citation network (**Figure 5B**), journals with at least 50 co-citations were selected, showcasing a prominent co-citation network among ‘Supportive Care in Cancer’, ‘Annals of Oncology’, and others.

**Table 2:** Top 10 journals and co-cited journals for research of CINV.

Rank	Journal	Counts	IF	Co-cited Journal	Co-citation	IF
1	Supportive care in cancer	119	3.1	Supportive care in cancer	3066	3.1
2	future oncology	20	3.3	Journal of Clinical Oncology	2210	45.3
3	Annals of oncology	19	50.5	Annals of oncology	1759	50.5
4	Pediatric blood & cancer	17	3.2	CANCER-AM CANCER SOC	709	6.2
5	Critical reviews in oncology hematology	12	6.2	New England Journal of Medicine	564	158.5
6	Medicine	12	1.6	European Journal of Cancer	389	8.4
7	Oncologist	12	5.8	European Journal of Pharmacology	379	5.0
8	International journal of clinical oncology	11	3.3	Lancet Oncology	372	51.1
9	Clinical journal of oncology nursing	10	1.1	Journal of Pain and Symptom Management	359	4.7
10	Journal of Oncology Pharmacy Practice	10	1.3	Journal of Supportive Oncology	344	–

**Figure 6:**
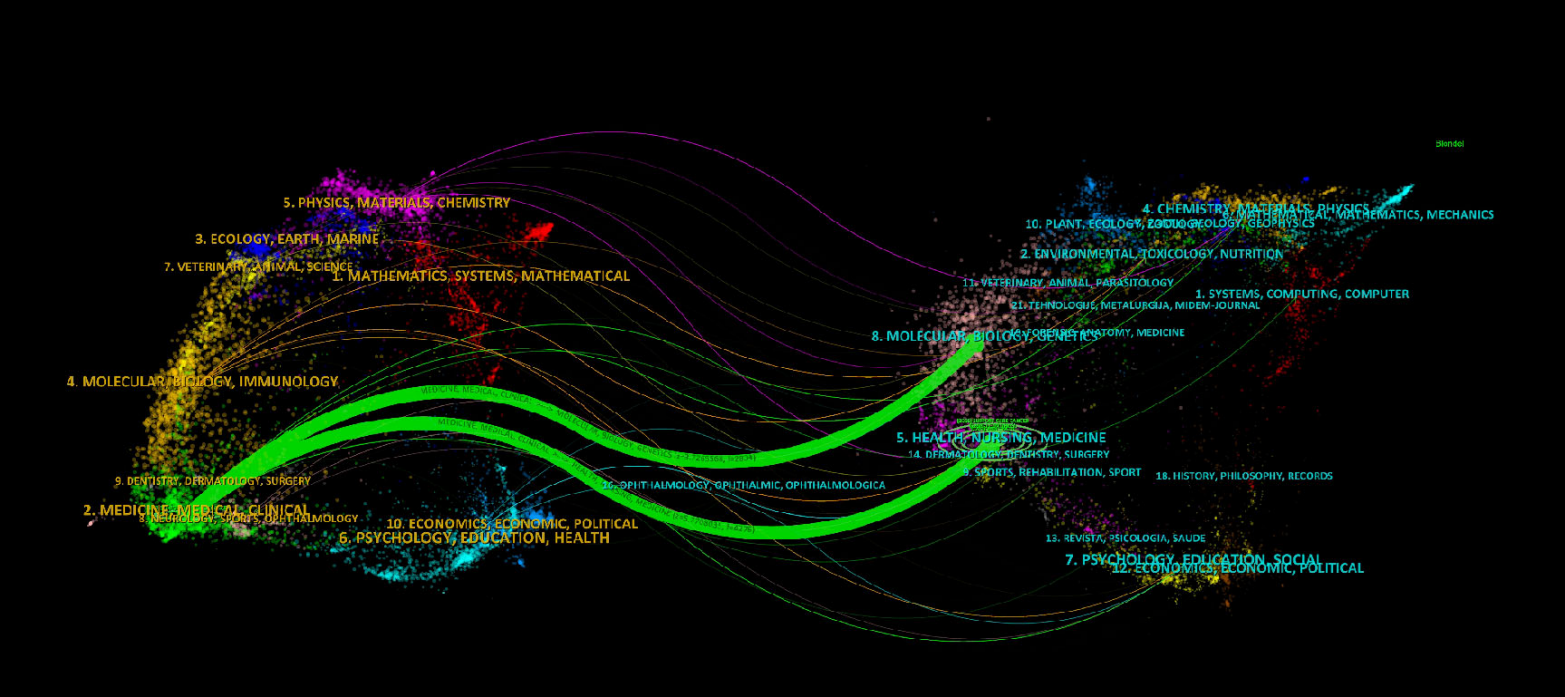
The dual-map overlay of journals on the research of CINV.

Furthermore, the dual map overlay of journals (**Figure 6**) illustrates citation dynamics, with clusters of citing journals on the left and cited journals on the right (19). The green path in **Figure 6** represents the main citation flow, indicating that research in Health/Nursing/Medicine journals is primarily cited by works in Medicine/Medical/Clinical journals.

### 3.4 Authors and co-cited authors

**Figure 7:**
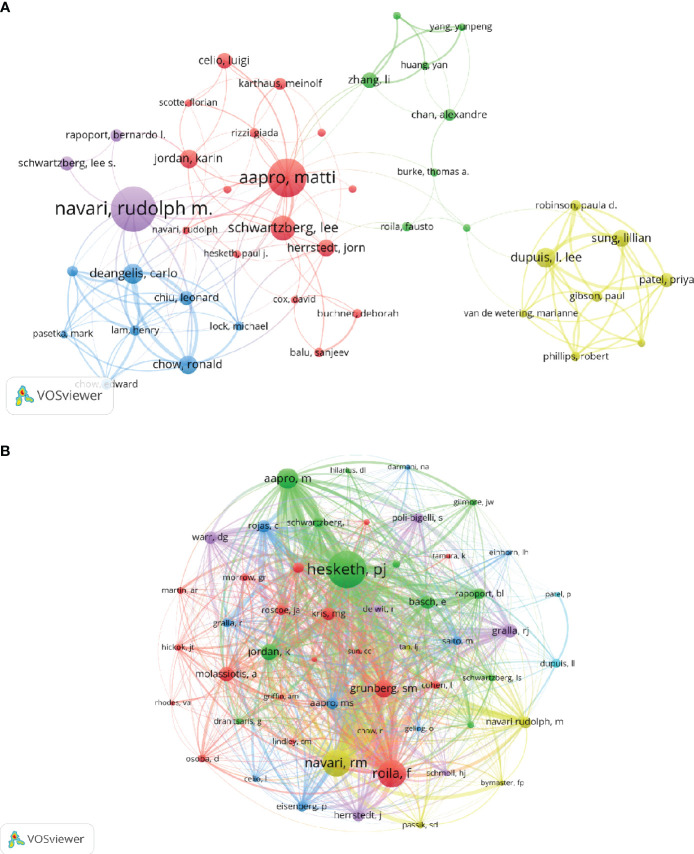
The visualization of authors and co-cited authors on the research of CINV.

A considerable total of 3,917 authors have made contributions to the research field of CINV. Among the most prolific, the top 12 authors include four who have each published over 15 papers (**Table 3**). To visualize the collaborative dynamics, we created a network diagram featuring authors who have authored five or more papers (**Figure 7A**). In this network, the nodes representing Rudolph M. Navari, Matti Aapro, Mototsugu Shimokawa, and Lee Schwartzberg are notably large, highlighting their substantial contributions to the field. Additionally, the diagram reveals tight collaborations among several authors, such as the close working relationship between Matti Aapro, Rudolph M. Navari, Lee Schwartzberg, among others.

**Table 3:** Top 12 authors and co-cited authors for research of CINV.

Rank	Authors	Counts	Rank	Co-CitedAuthors	Co-CitedAuthors
1	Rudolph M Navari	29	1	Paul J. Hesketh	995
2	Matti Aapro	25	2	Fausto Roila	621
3	Shimokawa Mototsugu	19	3	Rudolph M Navari	612
4	Lee Schwartzberg	16	4	Matti Aapro	395
5	Carlo DeAngelis	13	5	Steven M Grunberg	316
6	L. Lee Dupuis	13	6	Karin Jordan	282
7	Ronald Chow	12	7	Alex Molassiotis	251
8	Toshinobu Hayashi	12	8	Richard J. Gralla	224
9	Karin Jordan	12	9	Ethan Basch	207
10	Jorn Herrstedt	11	10	Mark G Kris	197
10	Toshiaki Saeki	11	11	Christine Rojas	193
10	Lillian Sung	11	12	David G Warr	191

In terms of co-citations, out of 6,722 authors, 12 have been co-cited more than 190 times (**Table 3**). The top co-cited author is PJ Hesketh (n=995), followed by F Roila (n=621) and RM Navari (n=612). We generated a co-citation network diagram for authors with at least 50 co-citations (**Figure 7B**). This diagram in **Figure 7B** illustrates the active co-citation collaborations among authors, indicating significant interactions, for example, between PJ Hesketh and F Roila, RM Navari, and M Aapro.

### 3.5 Co-cited references

**Figure 8:**
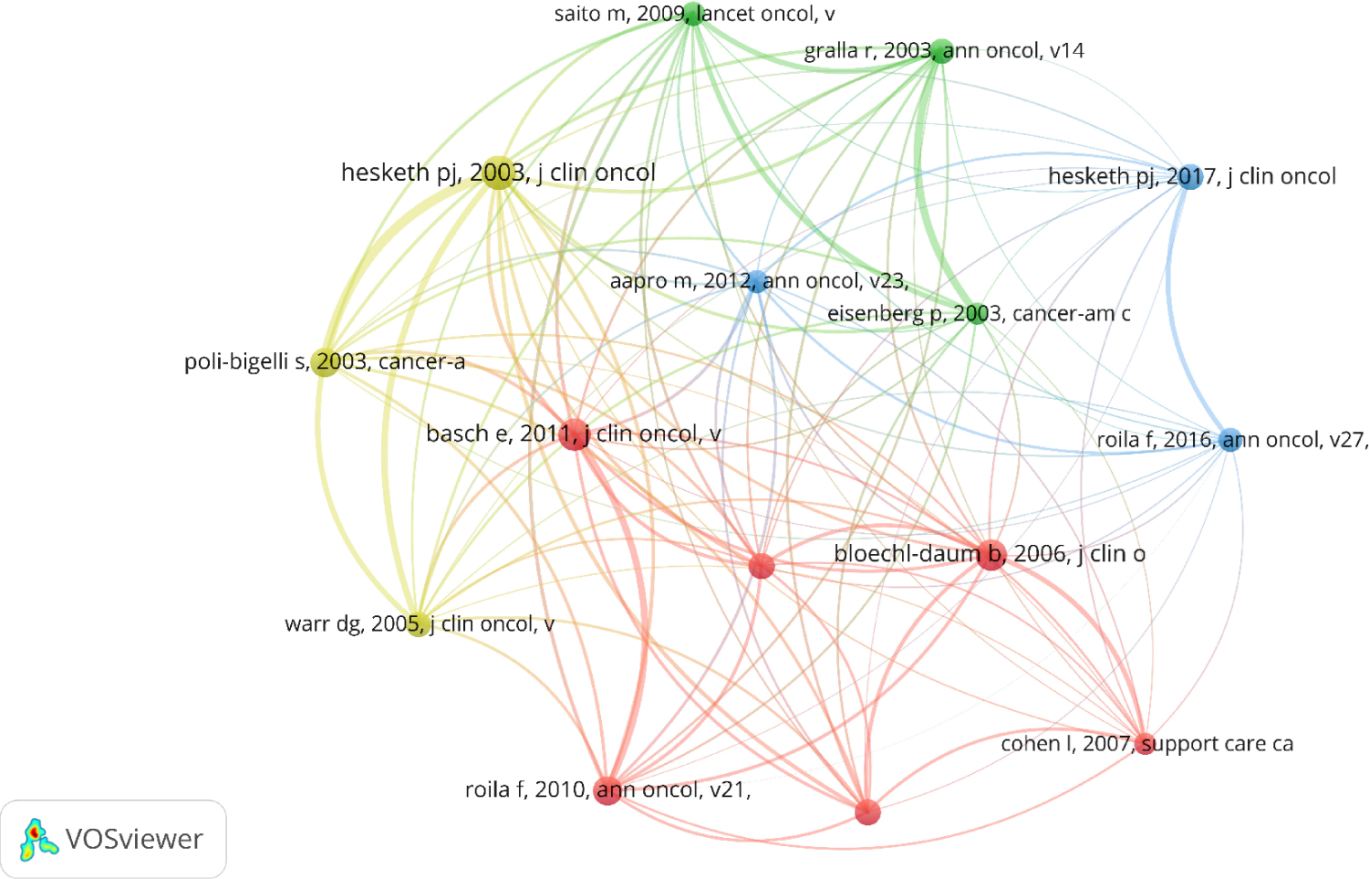
The visualization of co-cited references on the research of CINV.

Over the past two decades, research on CINV has been referenced in 9788 publications. The top 15 co-cited publications (**Table 4**) have each been cited at least 100 times, with three of them cited over 150 times (20–22). We constructed a co-citation network diagram with publications that have 50 or more co-citations (**Figure 8**). From **Figure 8**, it is evident that there is a significant co-citation relationship between “Hesketh PJ, 2003, J Clin Oncol” and publications like “Basch E, 2011, J Clin Oncol”, “Bloechl-Daum B, 2006, J Clin Oncol”, “Poli-Bigelli S, 2003, Cancer-Am Cancer Soc”, and “Roila F, 2010, Ann Oncol.”

**Table 4:** Top 15 co-cited references on research of CINV.

Rank	Co-cited reference	Citations
1	Hesketh PJ, 2003, j clin oncol, v21, p4112 (20)	171
2	Basch E, 2011, j clin oncol, v29, p4189 (21)	157
3	Bloechl-daum B, 2006, j clin oncol, v24, p4472 (22)	151
4	Roila F, 2010, ann oncol, v21, pv232 (23)	143
5	Poli-bigelli S, 2003, cancer-am cancer soc, v97, p3090 (24)	141
6	Grunberg SM, 2004, cancer-am cancer soc, v100, p2261 (25)	125
7	Hesketh PJ, 2008, new engl j med, v358, p2482 (26)	125
8	Hesketh PJ, 2017, j clin oncol, v35, p3240 (27)	124
9	Gralla R, 2003, ann oncol, v14, p1570 (28)	119
10	Warr DG, 2005, j clin oncol, v23, p2822 (29)	119
11	Roila F, 2016, ann oncol, v27, pv119 (30)	115
12	Saito M, 2009, lancet oncol, v10, p115 (31)	115
13	Aapro M, 2012, ann oncol, v23, p1986 (32)	109
14	Eisenberg P, 2003, cancer-am cancer soc, v98, p2473 (33)	104
15	Cohen I, 2007, support care cancer, v15, p497 (34)	103

### 3.6 Reference with citation bursts

**Figure 9:**
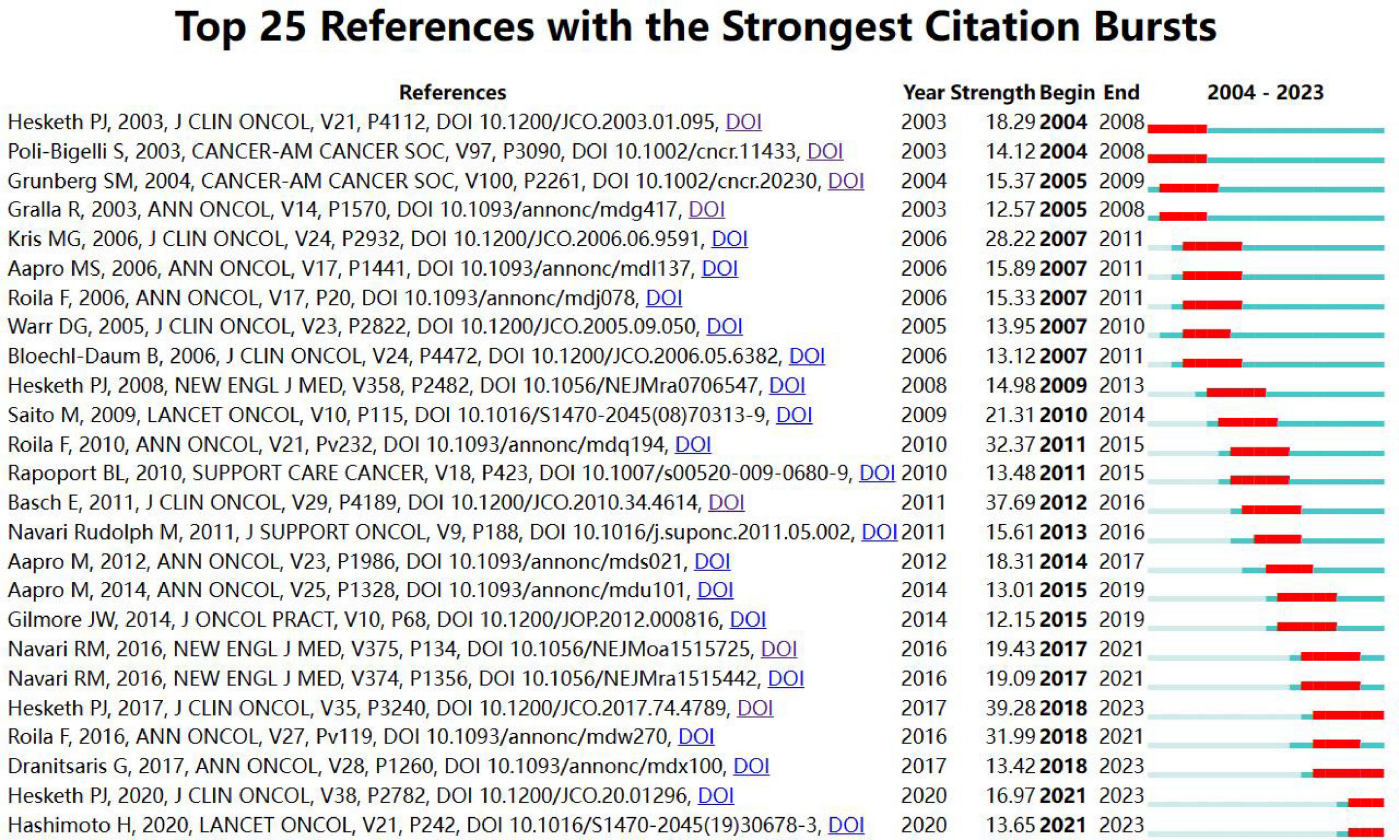
Top 25 references with strong citation bursts.

A citation burst in a reference indicates that the publication has experienced a significant increase in citations over a specific time frame, highlighting its influence and relevance in a particular field during that period. In our analysis, CiteSpace was utilized to perform a burst analysis on references, focusing on the top 50 with the highest burst strengths, as illustrated in **Figure 9**. The earliest citation bursts among these references began in 2004, while the most recent ones were observed in 2023 (5, 7, 20–32, 35–44).

The publication with the most pronounced citation burst (strength=39.28) is authored by Hesketh PJ et al., entitled “Antiemetics: American Society of Clinical Oncology Clinical Practice Guideline Update”. This paper’s burst period extended from 2018 to 2023. The burst strength of 25 significant publications ranged from 12.57 to 39.28, with their periods of influence lasting from 2 to 5 years.

### 3.7 Keyword co-occurrence, hotspots and frontiers

**Figure 10:**
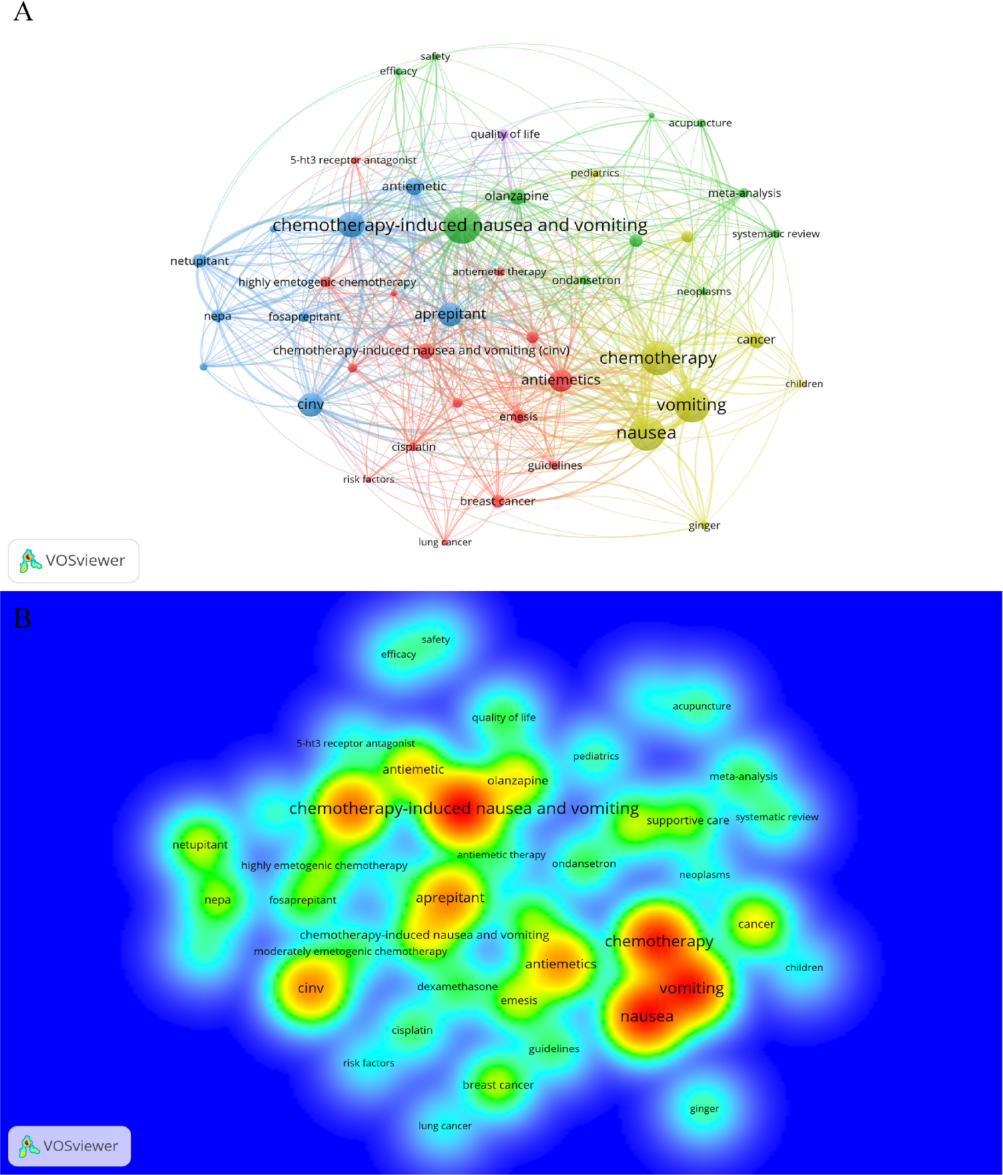
Keyword cluster analysis and the density map of the distribution of each keyword.

Keywords are pivotal in highlighting the focal points and directions of research in a specific area. In our study, we employed VOSviewer for the analysis of keyword co-occurrence within the field, selecting those that appeared ten times or more for cluster analysis using VOSviewer (**Figure 10A**). The density of lines between nodes in the visualization indicates the strength of the relationship between keywords. **Figure 10A** reveals four distinct clusters, each representing different research directions. The green cluster encompasses terms like ‘chemotherapy-induced nausea and vomiting’ and ‘olanzapine’. The red cluster includes ‘antiemetics’, ‘chemotherapy-induced nausea and vomiting (CINV)’, and ‘emesis’. The blue cluster features ‘palonosetron’, ‘aprepitant’, ‘CINV’, and ‘netupitant’. The yellow cluster is composed of ‘chemotherapy’, ‘vomiting’, ‘nausea’, and ‘cancer’.

Furthermore, the spatial distribution of each keyword is demonstrated in a density map (**Figure 10B**). After consolidating synonymous keywords such as ‘chemotherapy-induced nausea and vomiting’, ‘CINV’, and ‘antiemetic’/’antiemetics’, we compiled the top 17 most frequently co-occurring keywords, as shown in **Table 5**. This analysis indicates ‘chemotherapy-induced nausea and vomiting’ (n=338) as the most frequently occurring keyword, followed by ‘nausea’ (n=183), ‘vomiting’ (n=178), ‘chemotherapy’ (n=175), and ‘antiemetics’ (n=146), underscoring the primary areas of focus in this field of research.

**Table 5:** Top 17 keywords on research of CINV.

Rank	Keywords	Counts	Rank	Keywords	Counts
1	chemotherapy-induced nausea and vomiting	338	10	emesis	37
2	nausea	183	11	netupitant	37
3	vomiting	178	12	nausea and vomiting	35
4	chemotherapy	175	13	breast cancer	34
5	antiemetics	146	14	nepa	31
6	palonosetron	111	15	supportive care	30
7	aprepitant	94	16	granisetron	28
8	olanzapine	50	17	highly emetogenic chemotherapy	26
9	cancer	47			

**Figure 11:**
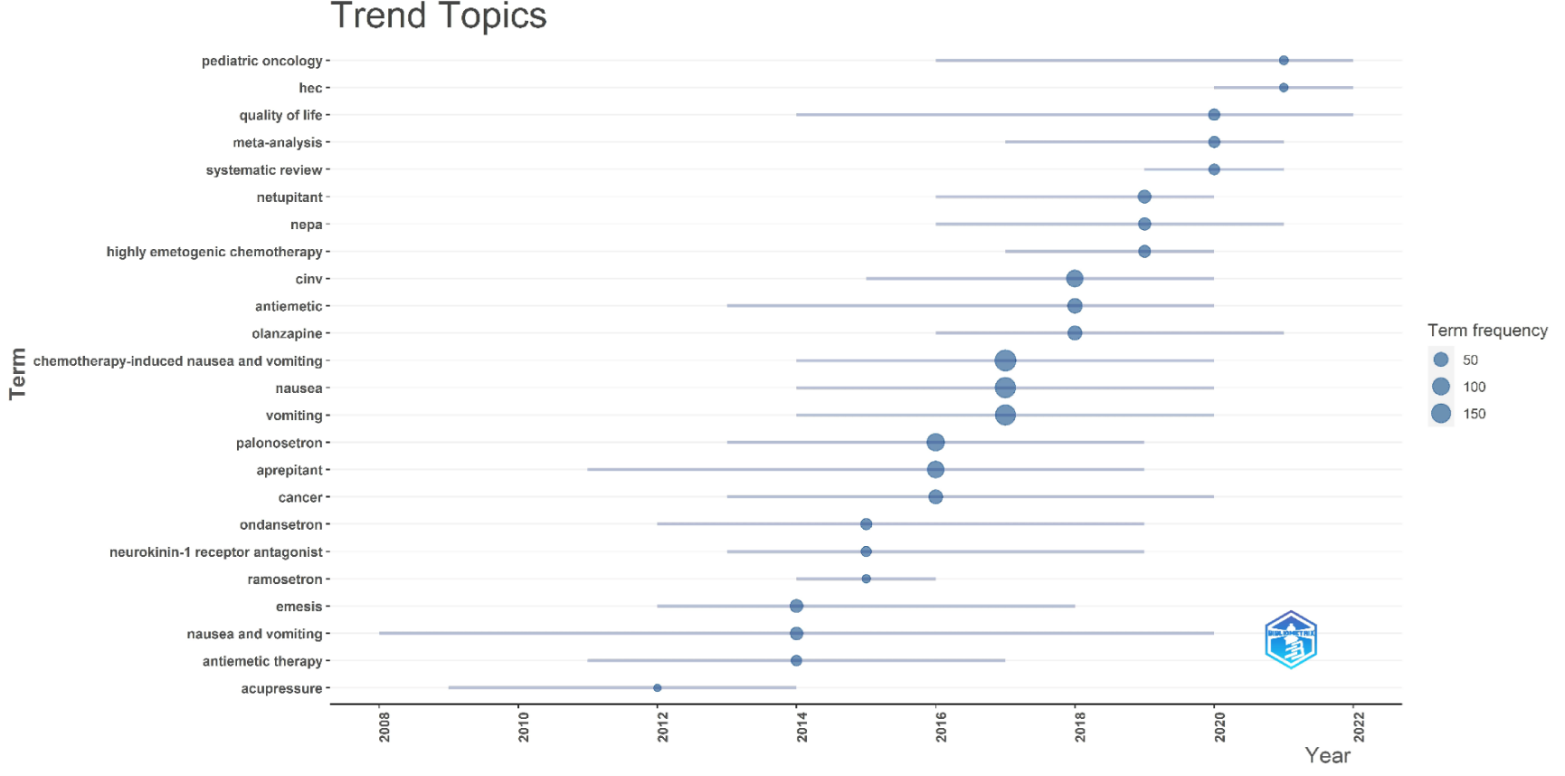
Trend topic analysis.

The keyword trend theme analysis (**Figure 11**) shows that from 2004 to 2014, there was less research on CINV, with studies mainly focused on specific symptoms and symptomatic treatments. The primary keywords during this period were antiemetic therapy, nausea and vomiting, and emesis. The years 2015-2019 marked a concentrated surge in CINV-related research, as studies delved deeper into the mechanisms of CINV occurrence. During this period, the application of drugs for treating or preventing CINV expanded significantly in clinical settings, particularly focusing on 5-HT receptor antagonists and NK-1 receptor antagonists. This era was marked by a surge in original clinical research, with key terms such as ramosetron, neurokinin-1 receptor antagonist, ondansetron, aprepitant, palonosetron, chemotherapy-induced nausea and vomiting, olanzapine, highly emetogenic chemotherapy, nepa, netupitant, and others gaining prominence. In the last three years (2020-2023), there has been a relative decrease in original research, primarily focusing on systematic reviews and meta-analyses. This trend may indicate certain bottlenecks in CINV research, with no significant breakthroughs in related basic theory, leading to fewer clinical studies. On the other hand, it also suggests that there is substantial potential for future research in CINV, requiring more scientists to dedicate their efforts towards achieving greater breakthroughs to benefit more patients.

## 4 Discussion

### 4.1 General information

This study collected a total of 734 publications. An examination of the yearly publication volume over the past twenty years shows a marked increase in the number of papers published in the latest decade compared to the one before it. Yet, as depicted in **Figure 2A**, there hasn’t been a discernible upward trajectory in the annual volume of publications in the last ten years. This suggests that research in CINV might not have garnered sufficient attention in recent years. This lack of escalating research interest could be contributing to the less effective management of CINV. Therefore, research related to CINV must receive more focus and recognition, also highlighting the untapped potential in this area of study.

### 4.2 Contributions by countries, institutions, journals, and authors

Among all countries involved in CINV-related research, the United States and China stand out as the top two contributing countries. The combined total of publications from these nations accounts for more than half of the overall output in this field, reaching an impressive 51.2%. Although the overall cancer incidence rate in the United States has been declining since the 1990s (45), advancements in cancer treatment have led to prolonged survival and lower mortality rates among cancer patients. Several factors have led to a consistent rise in the total number of cancer survivors in the United States, along with the country’s prominent role in the medical field. This has positioned the U.S. as the leading contributor to research in Chemotherapy-Induced Nausea and Vomiting (CINV), surpassing other nations. Meanwhile, China, with its vast population and a rising incidence of new cancer cases in recent years (35), has experienced an increase in patients needing chemotherapy. This surge has resulted in a substantial amount of CINV-related research, making China the second-largest contributor in this area, following the United States.

Regarding institutional contributions to research on CINV, the University of Toronto in Canada holds the leading position. The reason this institution leads, despite being in Canada and not the U.S., is due to the greater number of American institutions involved in CINV research, which are more dispersed compared to Canada. Among the top ten ranked institutions, five are in the U.S., leading to a lower publication count for individual American institutions compared to the University of Toronto. However, U.S. institutions actively collaborate with those in Canada, Switzerland, China, and the United Kingdom.

The top ten journals featuring articles on Chemotherapy-Induced Nausea and Vomiting (CINV) predominantly originate from the United States, with six American journals, three British, and one Japanese. These countries, particularly the United States, have been instrumental in propelling advancements in this field. A deeper analysis of the publication volume and the author collaboration network indicates that Rudolph M. Navari is a leading figure in CINV research, having authored the most papers in this area. His work extensively covers the prevention and treatment of CINV (5, 46), including detailed studies on the clinical use of 5-HT antagonists (47, 48), NK-1 antagonists (48), and olanzapine (36, 49, 50). Notably, Paul J Hesketh, who serves in various distinguished roles including the Director of the Lahey Health Cancer Institute, Director of the Sophia Gordon Cancer Center, Director of Thoracic Oncology at Lahey Hospital & Medical Center, and Professor of Medicine at Tufts University School of Medicine, has a primary focus on lung cancer. Nevertheless, he has made significant contributions to several CINV-related guidelines (27, 37), marking substantial achievements in this area and leading with 995 co-citations.

Continuing observation of the top ten authors by publication volume reveals that they are affiliated with different research institutions spread across North America, Europe, and Japan in Asia. This diversity indicates that CINV-related research has received significant attention globally.

### 4.3 Correlation analysis of keyword

The analysis of keyword co-occurrence, clustering, and burst detection provides valuable insights into the current research hotspots and frontiers in the study of Chemotherapy-Induced Nausea and Vomiting (CINV). The primary areas of focus in this field include:

#### 4.3.1 Symptoms and mechanisms of CINV

Chemotherapy-Induced Nausea and Vomiting (CINV) is classified into five categories: acute nausea and vomiting, delayed nausea and vomiting, breakthrough nausea and vomiting, refractory nausea and vomiting, and anticipatory nausea and vomiting (51–55). A corresponding table (5) (**Table 6**) has been created to categorize these types.

**Table 6:** Classification of CINV.

Classification	Definition
Acute	Manifests within the first 24 hours post-chemotherapy initiation, typically peaking around 5 to 6 hours.
Delayed	Develops 24 hours after chemotherapy and can persist for several days, usually from day 2 to day 5.
Breakthrough	Arises even with proper prophylactic treatment in place.
Anticipatory	Emerges before treatment, as a conditioned response due to previous experiences of CINV.
Refractory	Recurs in subsequent therapy cycles, distinct from anticipatory CINV.

Moreover, antineoplastic drugs are categorized into four levels based on the percentage of patients who experience acute vomiting in the absence of prophylactic measures. This classification system, which is crucial for guiding antiemetic therapy, defines the emetogenic potential of these drugs (38) as follows. High Emetogenicity: More than 90% likelihood of inducing acute vomiting without prophylaxis. Moderate Emetogenicity: Between 30% and 90% likelihood of causing acute vomiting in the absence of prophylaxis. Low Emetogenicity: A 10% to 30% chance of leading to acute vomiting without preventive measures. Minimal Emetogenicity: Zero to less than 10% probability of inducing acute vomiting without prophylaxis. This categorization is essential for healthcare professionals to effectively predict and manage the risk of CINV in patients undergoing chemotherapy.

Over the past three decades, there has been remarkable advancement in comprehending the mechanisms behind nausea and vomiting induced by chemotherapy drugs. Central to these mechanisms are neurotransmitters like dopamine, serotonin, and Substance P, which are key mediators in the vomiting process initiated by chemotherapy, with their receptors playing a significant role in this pathophysiological process (56). The response of vomiting due to chemotherapy can be traced through two primary pathways. Peripheral Pathways: These are activated within the first 24 hours of initiating chemotherapy, correlating with acute vomiting. Chemotherapy drugs stimulate enterochromaffin cells in the gastrointestinal tract to release serotonin, which then activates 5-HT receptors. This activation sends signals to the brain, initiating the vomiting reflex. Central Pathways: Activated after 24 hours of chemotherapy, these pathways are mainly associated with delayed vomiting. Neurotransmitter receptors in the brain’s posterior region are activated by chemotherapy drugs, leading to nausea and vomiting. Substance P activates neurokinin-1 (NK1) receptors in these pathways (51–55). Chemotherapy drugs activate neurotransmitter receptors in the brain’s posterior region, causing nausea and vomiting. These receptors, located near enterochromaffin cells in the gut, transmit chemotherapy-induced signals to the brainstem via the vagal afferents. Upon receiving signals, the brainstem processes the vomiting reflex and initiates the physical response of vomiting. Chemotherapy drugs stimulate the release of serotonin from enterochromaffin cells in the gut, activating 5-HT receptors that relay the signals to the brain. Substance P acts by activating neurokinin-1 (NK1) receptors (51–55). Furthermore, chemotherapy-induced nausea and vomiting can be influenced by operant conditioning, where environmental factors may provoke these symptoms (5).

#### 4.3.2 5-HT receptor antagonists

5-Hydroxytryptamine (5-HT) receptor antagonists function by inhibiting 5-HT3 receptors located in both the brain and the gastrointestinal tract. This action effectively reduces the nausea and vomiting induced by CINV. The release of 5-HT during chemotherapy activates 5-HT3 receptors, triggering nausea and vomiting. 5-HT antagonists effectively control CINV by inhibiting this process (26, 30). Multiple randomized controlled trials (RCTs) have demonstrated the effectiveness of various 5-HT receptor antagonists in treating CINV, including Ondansetron (57, 58), Granisetron (59), Tropisetron (60), and Palonosetron (28, 31).

#### 4.3.3 NK-1 receptor antagonists

NK-1 receptor antagonists work by blocking Neurokinin-1 (NK-1) receptors, effectively alleviating CINV. These receptors are widespread in the brain’s vomiting center and the gastrointestinal tract. Activation of NK-1 receptors promotes vomiting, especially after chemotherapy drug use. NK-1 receptor antagonists minimize the occurrence of nausea and vomiting by inhibiting specific receptors (26, 30). Various agents in this class, including Aprepitant (24), Fosaprepitant (61), Rolapitant (62), and Netupitant (39, 63), have been proven effective in multiple randomized controlled trials (RCTs). These drugs are commonly used in conjunction with 5-HT3 receptor antagonists and corticosteroids (such as dexamethasone) to improve therapeutic efficacy.

#### 4.3.4 Olanzapine

Olanzapine’s effectiveness in managing Chemotherapy-Induced Nausea and Vomiting (CINV) is attributed to its ability to antagonize multiple neurotransmitter receptors. As an atypical antipsychotic, Olanzapine, in its role against CINV, functions by blocking multiple receptors in the central nervous system. This includes antagonism of dopamine D2 receptors and 5-Hydroxytryptamine (5-HT2) receptors, histamine receptors, and acetylcholine receptors, all of which play crucial roles in the vomiting reflex (64, 65). Olanzapine, through its blockade of these receptors, effectively mitigates nausea and vomiting. This makes it particularly useful in cases where standard treatments for CINV— such as 5-HT3 and NK-1 receptor antagonists, along with corticosteroids — prove to be ineffective or insufficient. Its efficacy has also been validated in several authoritative clinical trials (36, 40, 49, 66).

### 4.4 Correlation analysis of co-cited references

Through the analysis of highly cited publications, key knowledge sources within a field can be identified. In our study, we concentrated on the top three cited references to acquire a comprehensive understanding of the subject matter. Each of these three articles has been cited more than 150 times, making them highly representative and significant in the realm of the study.

The first article titled “The oral neurokinin-1 antagonist aprepitant for the prevention of chemotherapy-induced nausea and vomiting: a multinational, randomized, double-blind, placebo-controlled trial in patients receiving high-dose cisplatin–the Aprepitant Protocol 052 Study Group” (20) is to assess if the Aprepitant regimen outperforms the standard treatment in preventing CINV. In the study, the control group was administered the standard regimen, which included ondansetron and dexamethasone on the first day, followed by dexamethasone alone on days 2 to 4. Conversely, the experimental group received the Aprepitant regimen: a combination of aprepitant, ondansetron on day 1, aprepitant on days 2 to 3, and dexamethasone on day 1 to 4. The study’s findings indicated that the Aprepitant group experienced a significantly higher rate of complete relief from nausea and vomiting from day 1 to day 5 compared to the standard treatment group, and the regimen was well-tolerated.

The second article titled “Antiemetics: American Society of Clinical Oncology Clinical Practice Guideline Update” (21) reviews the literature comprehensively, including the Cochrane Library, MEDLINE, and ASCO conference materials and various international cancer support treatment associations, to study the complete remission rate and incidence of CINV to update the ASCO Antiemetics guidelines. The conclusions include the reclassification of anthracycline and cyclophosphamide combination treatment as a high-emetic regimen. In the treatment of patients receiving high-emetic chemotherapy regimens, or any regimen with a high risk of inducing nausea and vomiting, the recommended approach includes a combination of a 5-HT receptor antagonist, an NK-1 receptor antagonist, and dexamethasone. For those undergoing high emetic risk radiation therapy, the recommendation is to administer a 5-HT (3) receptor antagonist prior to each fraction of radiation and maintain this for 24 hours following the treatment. Moreover, administering dexamethasone for a 5-day period, specifically during the first to fifth fractions, could prove advantageous. For chemotherapy regimens with a moderate risk of emesis, the combination of palonosetron and dexamethasone is suggested. For treatments involving low-risk drugs, administering dexamethasone prior to the first chemotherapy session is an option for patients. The update committee has also highlighted the critical need for continuous symptom monitoring throughout the course of treatment. It has been observed that clinicians frequently underestimate the occurrence of nausea, which is often not as effectively managed as vomiting.

The third article titled “Delayed nausea and vomiting continue to reduce patients’ quality of life after highly and moderately emetogenic chemotherapy despite antiemetic treatment” (22), is a prospective, multicenter, international study. This research compares the effects of acute (occurring within 24 hours of chemotherapy) and delayed (manifesting 2-5 days post-chemotherapy) Chemotherapy-Induced Nausea and Vomiting (CINV) on the QoL of patients undergoing and MEC, respectively.

The study’s findings highlight a significant issue: even in cases where patients did not suffer from nausea and vomiting in the initial 24 hours following moderately emetogenic chemotherapy, their quality of life was still adversely impacted by CINV in subsequent days. Notably, the study reveals that nausea had a more detrimental effect on patients’ daily life compared to vomiting.

### 4.5 Limitations

Our thorough investigation of articles on CINV from 2004 to 2023 has provided valuable insights, yet there are notable limitations to be acknowledged as follows: Data Collection Cut-off Date: The articles were compiled as of October 5, 2023, covering the period from January 1, 2004, to September 30, 2023. As the year was not complete at the time of data collection, there may be additional relevant articles published after this date that were not included in our analysis. Database Limitation: Considering that WOS assigns document type labels more accurately than other databases such as Scopus (67), our research solely utilized the Web of Science database for sourcing articles. This focus may have resulted in the exclusion of pertinent articles available in other academic databases, potentially narrowing the scope of our review. Language Restriction: The study was confined to articles and reviews published in English. This restriction means that significant contributions to the field of CINV, published in other languages, might not have been considered, possibly overlooking important global perspectives and findings.

These limitations suggest that while our study provides a comprehensive overview, it may not encapsulate the complete global research landscape on CINV, and there could be additional valuable insights and data in publications beyond our scope.

## 5 Conclusions

This study stands as the inaugural systematic analysis of Chemotherapy-Induced Nausea and Vomiting (CINV)-related literature using bibliometric and knowledge mapping methodologies. To enhance the depth and breadth of our analysis, we employed advanced tools such as CiteSpace, VOSviewer, and the R package “bibliometrix”. These tools enabled us to glean a more comprehensive and varied set of insights from the data. Distinguishing itself from traditional reviews, this study offers a novel and objective perspective on the landscape of CINV research. Overall, in the past two decades, the number of publications in the most recent decade has significantly increased compared to the previous decade, but there hasn’t been a noticeable upward trend in annual publication volume in the last ten years. This suggests that research in this field may not have received sufficient attention recently, which could be one of the reasons for the less effective management of CINV. Therefore, more attention and focus are needed on CINV-related research, necessitating further studies in this area and enhanced collaboration between countries and institutions. Currently, the most active frontier research focuses mainly on the combined use of 5-HT receptor antagonists, NK-1 receptor antagonists, Olanzapine, and Dexamethasone, including their timing and dosages. We are confident that the results of this study will offer valuable implications for future studies.

## Data availability statement

The original contributions presented in the study are included in the article/supplementary material. Further inquiries can be directed to the corresponding author.

## Author contributions

S-CT: Data curation, Formal analysis, Methodology, Writing – original draft, Writing – review & editing. JY: Conceptualization, Investigation, Writing – review & editing. XL: Methodology, Software, Writing – original draft. R-XH: Data curation, Methodology, Writing – original draft. JC: Formal analysis, Methodology, Software, Writing – original draft, Writing – review & editing.
